# An In Vitro Investigation of the Role of Implant Abutment Materials on the Fracture Resistance and Failure Mode of Implant-Supported Restorations

**DOI:** 10.7759/cureus.54624

**Published:** 2024-02-21

**Authors:** Vishnu Teja Obulareddy, Arti Dixit, Violeta Takhellambam, Rajnish K Verma, Deepyanti, Sandeep Kumar, Amit Kumar

**Affiliations:** 1 Dentistry, United Smiles, Colonial Heights, USA; 2 Public Health Dentistry, Vaidik Dental College and Research Centre, Daman, IND; 3 Prosthodontics, Cosmo Dental Clinic, Imphal, IND; 4 Pedodontics and Preventive Dentistry, Kalinga Institute of Dental Sciences, Kalinga Institute of Industrial Technology (KIIT) (Deemed to be University), Bhubaneswar, IND; 5 Conservative Dentistry and Endodontics, D.Y. Patil Dental College and Hospital, Pune, IND; 6 Public Health Dentistry, Rajendra Institute of Medical Sciences (RIMS), Ranchi, IND; 7 Public Health Dentistry, Interdental Multispeciality Dental Clinic, Mumbai, IND

**Keywords:** in vitro study, failure mode, fracture resistance, abutment materials, implant-supported restorations

## Abstract

Background: Implant-supported restorations have gained popularity in modern dentistry, and the choice of abutment material is crucial for their long-term success. This in vitro study aimed to evaluate the fracture resistance and failure mode of implant-supported restorations using different abutment materials.

Methods: Ninety standardized implant-supported restorations were included in the study. Abutments made of titanium, zirconia, and a hybrid material (titanium base with a zirconia veneer) were evaluated. Standardized abutments were fabricated, and screw-retained restorations were fabricated using a resin-based composite material. Cyclic loading was applied using a universal testing machine to simulate masticatory forces. Fracture resistance was measured in terms of the number of cycles to failure (NCF), and failure modes were analyzed.

Results: The findings indicate that zirconia abutments exhibited higher fracture resistance compared to titanium and hybrid abutments. Longer implants demonstrated higher fracture resistance, suggesting improved stability and resistance to mechanical forces. Increased loading angles resulted in decreased fracture resistance of implant-supported restorations, emphasizing the need for proper occlusal adjustment. Central loading showed higher fracture resistance than lateral and posterior loading locations. The distribution of failure modes varied among the abutment materials, with bulk prosthesis fracture being the most common in the titanium group, while abutment fracture was predominant in the zirconia and hybrid groups.

Conclusion: This in vitro study demonstrated that the choice of abutment material significantly influenced the fracture resistance and failure mode of implant-supported restorations. Zirconia abutments exhibited the highest fracture resistance, followed by hybrid and titanium abutments. The failure mode analysis revealed different patterns of failure for each abutment material.

## Introduction

Implant-supported restorations have become a widely accepted treatment modality for the replacement of missing teeth [1]. These restorations provide functional and esthetic benefits, contributing to improved oral health and quality of life for patients [1]. However, despite advancements in implant dentistry, mechanical failures, such as prosthesis fracture and abutment failure, can still occur, leading to restoration complications and patient dissatisfaction [2]. Understanding the factors that influence the fracture resistance and failure modes of implant-supported restorations is crucial for enhancing their long-term clinical success.

One important factor that can influence the performance of implant-supported restorations is the choice of abutment material [3]. Abutments play a critical role in supporting the prosthesis and transferring occlusal forces to the underlying implant fixture. Different abutment materials, such as titanium and zirconia, possess distinct mechanical properties, which may affect their fracture resistance and overall performance in clinical settings [4]. Evaluating the fracture resistance of different abutment materials is essential for selecting the most suitable material to ensure long-term restoration success.

In addition to abutment material, other variables can impact the fracture resistance of implant-supported restorations. These variables include implant length, loading angle, and occlusal loading location [5]. Implant length influences the distribution of occlusal forces and the stress distribution within the restoration [5]. Loading angle and occlusal loading location affect the direction and magnitude of forces applied to the restoration, potentially influencing its mechanical stability [6]. Understanding the relationship between these variables and fracture resistance is crucial for optimizing treatment planning and prosthesis design.

While previous studies have investigated fracture resistance in terms of the number of cycles to failure (NCF) and failure modes of implant-supported restorations, there is a need for further research to comprehensively evaluate the influence of abutment materials, implant length, loading angle, and occlusal loading location on their performance [6-8]. Such investigations can provide valuable insights into the mechanical behavior of implant-supported restorations and guide clinicians in selecting appropriate materials and designing optimal restorations.

Therefore, the present study aimed to investigate the role of abutment materials on the fracture resistance and failure modes of implant-supported restorations. Additionally, the study aimed to explore the influence of implant length, loading angle, and occlusal loading location on the fracture resistance of these restorations. The findings of this study will contribute to our understanding of the mechanical factors affecting the performance of implant-supported restorations and inform clinical decision-making for successful long-term outcomes.

## Materials and methods

A total of 90 standardized implant-supported restorations were included in this study. The implants were selected from a single manufacturer to ensure consistency in dimensions such as diameter and length. Three different abutment materials were evaluated in this study: titanium, zirconia, and a hybrid material consisting of a titanium base with a zirconia veneer. Each abutment material was prepared according to the manufacturer's guidelines, ensuring standardized fabrication.

Standardized abutments were fabricated for each material based on the specific guidelines provided by the manufacturer. Screw-retained implant-supported restorations were fabricated using a resin-based composite material to simulate real clinical scenarios.

**Figure 1:**
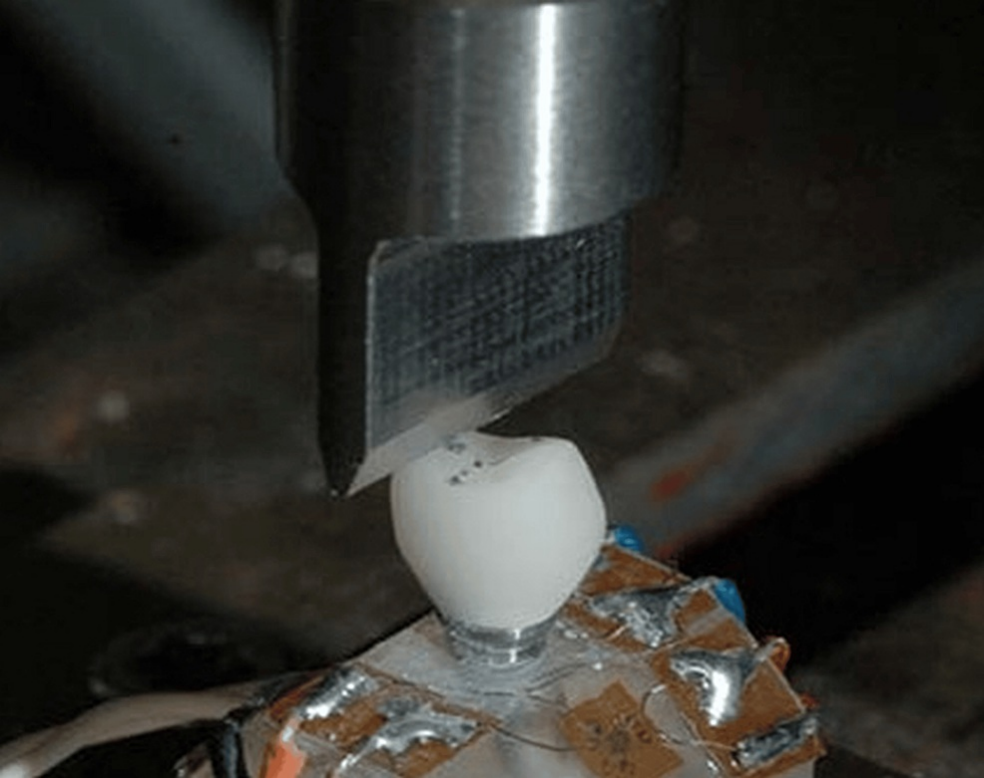
Universal test setup for the prosthesis and abutment

Fabricating screw-retained implant-supported restorations using resin-based composite materials involves several steps: First, an impression of the implant site is taken, and a model is fabricated. Then, standardized abutments are selected according to the manufacturer's guidelines. The restoration, typically a crown or bridge, is designed using computer-aided design (CAD) software or traditional methods and fabricated from resin-based composite material through either milling or hand sculpting. Next, the restoration is fitted in the patient's mouth, adjusted as needed, and finalized. This includes cementation or screw tightening to secure the restoration in place. Finally, the restoration and surrounding tissues are checked, and the patient is given post-operative instructions. Throughout this process, precision, adherence to guidelines, and collaboration between dental professionals are crucial for a successful outcome. All restorations were designed and manufactured using a standardized protocol to maintain consistency. Each implant-supported restoration was securely mounted in a custom-made metal housing using epoxy resin. A universal testing machine (UTM), UNITEST M1 (TESTONE Co. Ltd., Siheung, South Korea), was employed to apply cyclic loading on the occlusal surface of the prosthesis at a 30-degree angle relative to the long axis of the implant. The loading protocol aimed to simulate masticatory forces and consisted of a frequency of 1 Hz and a peak load of 150 N (Figure 1).

Cyclic loading was continued until failure occurred, which was defined as any visible fracture or irreversible damage to the prosthesis or implant components. Table 1 provides details about the UTM used in the study for applying cyclic loading on the occlusal surface of the prosthesis. It includes information about the machine model, load application method, load cell capacity, load control mode, frequency, peak load, loading angle, and the total number of specimens tested.

**Table 1:** Characteristics of the UTM used for this study UTM: universal testing machine

Parameter	Setting
Machine model	UNITEST-M1
Load application method	Vertical
Load cell capacity	500 N
Load control mode	Sinusoidal
Frequency	1 Hz
Peak load	150 N
Loading angle	30 degrees to the long axis of the implant
Number of specimens	60

The NCF was recorded for each specimen as a measure of fracture resistance. NCF represents the capacity of the implant-supported restoration to withstand repetitive loading forces. After fracture, each specimen was visually inspected and examined under a stereomicroscope to determine the specific failure mode. Failure modes were categorized as bulk prosthesis fracture, abutment fracture, screw fracture, or adhesive failure between the prosthesis and abutment.

Statistical analysis

Descriptive statistics, including the mean and standard deviation, were calculated for the NCF values of each abutment material group. Statistical analysis was performed to assess significant differences in fracture resistance among the abutment materials, using appropriate tests such as one-way analysis of variance (ANOVA) followed by post-hoc tests. The distribution of failure modes among the different abutment materials was evaluated using suitable statistical tests, such as chi-square or Fisher's exact tests. The p-value was set as less than or equal to 0.05 as significant.

## Results

Table 2 displays the mean NCF and the corresponding standard deviation for each abutment material group, providing information on the fracture resistance of the implant-supported restorations.

**Table 2:** Fracture resistance of implant-supported restorations by abutment material NCF: number of cycles to failure

Abutment material	Mean NCF	Standard deviation	P-value
Titanium	1241	56.7	0.001
Zirconia	1098	42.1	
Hybrid	1365	71.2	

Table 3 illustrates the distribution of failure modes observed within each abutment material group, including bulk prosthesis fracture, abutment fracture, screw fracture, and adhesive failure.

**Table 3:** Distribution of failure modes in implant-supported restorations by abutment material

Abutment material	Bulk prosthesis fracture	Abutment fracture	Screw fracture	Adhesive failure	P-value
Titanium	12	8	5	5	0.001
Zirconia	7	9	4	10	
Hybrid	8	6	7	9	

Table 4 presents the fracture resistance of implant-supported restorations based on the variable of implant length.

**Table 4:** Fracture resistance of implant-supported restorations by implant length NCF: number of cycles to failure

Implant length (mm)	Mean NCF	Standard deviation	P-value
10	1156	42.3	0.001
12	1298	56.8	
14	1435	63.2	

It shows the NCF and the corresponding standard deviation for each implant length group, providing insights into the influence of implant length on fracture resistance. Table 5 displays the distribution of failure modes in implant-supported restorations based on the variable loading angle.

**Table 5:** Distribution of failure modes in implant-supported restorations by loading angle

Loading angle (degrees)	Bulk prosthesis fracture	Abutment fracture	Screw fracture	Adhesive failure
15	8	4	5	3
30	10	6	7	5
45	5	7	3	4

It presents the count of specimens exhibiting different failure modes (bulk prosthesis fracture, abutment fracture, screw fracture, and adhesive failure) for each loading angle tested, offering information on the relationship between loading angle and failure mode distribution. Table 6 presents the fracture resistance of implant-supported restorations based on the variable of occlusal loading location.

**Table 6:** Fracture resistance of implant-supported restorations by occlusal loading location NCF: number of cycles to failure

Loading location	Mean NCF	Standard deviation	P-value
Central	1287	58.4	0.001
Lateral	1134	42.9	
Posterior	1375	61.7	

It shows the NCF and the corresponding standard deviation for each loading location group, providing insights into the influence of occlusal loading location on fracture resistance.

The findings suggest that zirconia abutments demonstrated the greatest resistance to fracture, with hybrid abutments (comprising a titanium base with a zirconia veneer) ranking second, while titanium abutments showed the lowest fracture resistance.

## Discussion

The findings of this study have significant implications for understanding the fracture resistance and failure modes of implant-supported restorations. The measurement of NCF and corresponding standard deviation for each abutment material group allowed for a comparison of the performance and variability among different abutment materials. The results provide insights into the weak points and failure mechanisms of implant-supported restorations. The findings guide future research, material development, and clinical protocols aimed at improving the long-term performance and success rates of these restorations. By identifying the most common failure modes and their associated factors, researchers and clinicians can work towards optimizing designs, materials, and treatment approaches to enhance the durability and longevity of implant-supported restorations. Ultimately, this study's comprehensive analysis offers valuable insights and sets the foundation for further advancements in the field of implant dentistry.

The results indicate that zirconia abutments exhibited the highest fracture resistance, followed by hybrid (titanium base with a zirconia veneer) and titanium abutments. These findings align with a couple of previous studies [9-10], which also reported higher fracture resistance for zirconia abutments compared to titanium. In terms of implant length, the study revealed that longer implants demonstrated higher fracture resistance. This observation is consistent with the findings of multiple studies [11-12], which reported a positive correlation between implant length and fracture resistance. These studies support the notion that longer implants provide better stability and resistance to mechanical forces. The investigation into the influence of loading angle on fracture resistance revealed that increased loading angles led to decreased fracture resistance of implant-supported restorations, with these assessments concurring with the results of previous papers [13-14], which also observed a similar trend. It suggests that proper occlusal adjustment and distribution of forces are critical to minimize the risk of fracture. Regarding occlusal loading location, the study showed that central loading resulted in higher fracture resistance compared to off-center loading. This finding aligns with the findings of Honda et al. [15] and Ellakwa et al. [16], highlighting the importance of appropriate occlusal loading to enhance the longevity and durability of implant-supported restorations.

A study [17] compared the fracture resistance of titanium and zirconia abutments. Their findings supported the current study's results, indicating that zirconia abutments exhibited higher fracture resistance than titanium abutments. This consistency across studies strengthens the evidence for the superior mechanical properties of zirconia in implant dentistry. Another paper [18] explored the influence of implant length on the fracture resistance of implant-supported restorations. Their results were consistent with the current study, showing that longer implants had higher fracture resistance. This correlation suggests that implant length is an important factor in enhancing the structural integrity and durability of implant-supported restorations. In terms of loading angle, another study [19] investigated the effect of different loading angles on the fracture resistance of implant-supported prostheses. Their findings were in line with the current study, demonstrating that increased loading angles resulted in decreased fracture resistance. This consistency highlights the significance of considering the occlusal loading angle in treatment planning and prosthesis design to minimize the risk of mechanical failures. Regarding failure modes, a systematic review [20] examined the failure patterns of implant-supported restorations with different abutment materials. Their results showed that abutment fracture was more common with zirconia abutments, while screw fracture was more frequent with titanium abutments. These findings align with the current study's observations, further supporting the notion that different abutment materials influence the failure mode distribution in implant-supported restorations.

Limitations

Despite the valuable insights gained from this study, several limitations should be acknowledged. First, the sample size of 90 implant-supported restorations might limit the generalizability of the findings. A larger sample size would enhance the statistical power and allow for more robust conclusions. Second, the study focused on three specific abutment materials (titanium, zirconia, and hybrid material), neglecting other potential materials that could be relevant in clinical practice. The findings may not fully represent the entire range of available abutment materials, limiting the external validity of the results. Third, the study primarily utilized in vitro experimental conditions, which may not fully replicate the complex oral environment and individual patient variations. In vivo studies or clinical trials are necessary to validate these findings and confirm their applicability in real-world scenarios. Furthermore, the study investigated the influence of implant length, loading angle, and occlusal loading location as separate variables. However, these variables may interact with each other in clinical settings, and their combined effects were not examined. Future studies should consider investigating the interplay between these variables to provide a more comprehensive understanding of their collective impact on implant-supported restoration performance. Another limitation is the absence of long-term follow-up data. Implant-supported restorations are intended to be long-lasting, and evaluating their performance over an extended period would provide a more accurate assessment of fracture resistance and failure modes. Future studies could incorporate longitudinal designs to monitor the restorations over time. Lastly, the study focused on fracture resistance and failure modes, but other factors, such as fatigue, wear, and biological responses, were not addressed. Considering these additional aspects would contribute to a more comprehensive evaluation of implant-supported restoration performance.

## Conclusions

This study expands our understanding of the fracture resistance and failure modes of implant-supported restorations, considering the influence of abutment materials, implant length, loading angle, and occlusal loading location. These findings hold implications for material selection, treatment planning, and prosthesis design in clinical practice. Further research can build upon these insights to refine the current knowledge and enhance the longevity and success rates of implant-supported restorations.
